# Mathematical modelling and phylodynamics for the study of dog rabies dynamics and control: A scoping review

**DOI:** 10.1371/journal.pntd.0009449

**Published:** 2021-05-27

**Authors:** Maylis Layan, Simon Dellicour, Guy Baele, Simon Cauchemez, Hervé Bourhy

**Affiliations:** 1 Mathematical Modelling of Infectious Diseases Unit, Institut Pasteur, UMR2000, CNRS, Paris, France; 2 Sorbonne Université, Paris, France; 3 Spatial Epidemiology Lab (SpELL), Université Libre de Bruxelles, Bruxelles, Belgium; 4 Department of Microbiology, Immunology and Transplantation, Rega Institute, KU Leuven, Leuven, Belgium; 5 Lyssavirus Epidemiology and Neuropathology Unit, Institut Pasteur, Paris, France; 6 WHO Collaborating Centre for Reference and Research on Rabies, Institut Pasteur, Paris, France; Environment and Sustainability Institute, UNITED KINGDOM

## Abstract

**Background:**

Rabies is a fatal yet vaccine-preventable disease. In the last two decades, domestic dog populations have been shown to constitute the predominant reservoir of rabies in developing countries, causing 99% of human rabies cases. Despite substantial control efforts, dog rabies is still widely endemic and is spreading across previously rabies-free areas. Developing a detailed understanding of dog rabies dynamics and the impact of vaccination is essential to optimize existing control strategies and developing new ones. In this scoping review, we aimed at disentangling the respective contributions of mathematical models and phylodynamic approaches to advancing the understanding of rabies dynamics and control in domestic dog populations. We also addressed the methodological limitations of both approaches and the remaining issues related to studying rabies spread and how this could be applied to rabies control.

**Methodology/principal findings:**

We reviewed how mathematical modelling of disease dynamics and phylodynamics have been developed and used to characterize dog rabies dynamics and control. Through a detailed search of the PubMed, Web of Science, and Scopus databases, we identified a total of *n* = 59 relevant studies using mathematical models (*n* = 30), phylodynamic inference (*n* = 22) and interdisciplinary approaches (*n* = 7). We found that despite often relying on scarce rabies epidemiological data, mathematical models investigated multiple aspects of rabies dynamics and control. These models confirmed the overwhelming efficacy of massive dog vaccination campaigns in all settings and unraveled the role of dog population structure and frequent introductions in dog rabies maintenance. Phylodynamic approaches successfully disentangled the evolutionary and environmental determinants of rabies dispersal and consistently reported support for the role of reintroduction events and human-mediated transportation over long distances in the maintenance of rabies in endemic areas. Potential biases in data collection still need to be properly accounted for in most of these analyses. Finally, interdisciplinary studies were determined to provide the most comprehensive assessments through hypothesis generation and testing. They also represent new avenues, especially concerning the reconstruction of local transmission chains or clusters through data integration.

**Conclusions/significance:**

Despite advances in rabies knowledge, substantial uncertainty remains regarding the mechanisms of local spread, the role of wildlife in dog rabies maintenance, and the impact of community behavior on the efficacy of control strategies including vaccination of dogs. Future integrative approaches that use phylodynamic analyses and mechanistic models within a single framework could take full advantage of not only viral sequences but also additional epidemiological information as well as dog ecology data to refine our understanding of rabies spread and control. This would represent a significant improvement on past studies and a promising opportunity for canine rabies research in the frame of the One Health concept that aims to achieve better public health outcomes through cross-sector collaboration.

## Author summary

Rabies is a fatal yet vaccine-preventable zoonotic disease. Domestic dog populations are known to constitute the predominant reservoir of rabies in developing countries, causing 99% of human rabies cases. Despite valuable efforts to control rabies spread, the last two decades have seen only a limited reduction in the global rabies disease burden. Dog rabies is still endemic in Africa, Asia, and the Middle East, in part due to remaining knowledge gaps on dog rabies dynamics. We conducted an in-depth review of phylodynamic approaches and mathematical models used to study the spread and control of rabies in domestic dogs. We identified 59 relevant studies which used mathematical models (30), phylodynamic approaches (22), or interdisciplinary approaches (7). Our study revealed that these approaches disentangled different aspects of rabies spread and control. Mathematical models support the role of dog population heterogeneity as a key driver of rabies spread, and the overwhelming efficacy of dog vaccination campaigns to control rabies. Phylodynamic studies confirm the role of frequent reintroduction events and human-mediated transportation over long distances in rabies maintenance. Interdisciplinary studies represent a powerful tool to generate and test hypotheses on rabies spread. Finally, we identified new avenues which represent a promising opportunity for canine rabies research to achieve more impactful public health outcomes.

## Introduction

### Background

Rabies is a viral zoonosis affecting the central nervous system of mammals that is almost always fatal to humans. Domestic dogs represent the main reservoir of rabies virus (RABV) worldwide. They are responsible for 99% of human rabies cases [1]. In-depth understanding of dog ecology and host-pathogen interactions is necessary to characterize rabies dynamics and design appropriate control measures. Rabies is a vaccine-preventable disease in both human and canine populations, and dog vaccination is the most cost-effective control measure [2]. Strong evidence is available for the efficacy of dog rabies elimination programs in endemic areas [3–7], notably in South America where massive dog vaccination campaigns in the 1980s alleviated the burden of canine rabies. Regardless, there has been only little improvement of the global burden since the successes in South America. Dog rabies is still endemic in Africa, Asia, and the Middle East [8,9].

In 2015, the World Health Organization (WHO), the Global Alliance for Rabies Control (GARC), the World Organization for Animal Health (OIE) and the Food and Agriculture Organization of the United Nations (FAO) launched a comprehensive framework targeting the global elimination of dog-mediated human rabies by 2030 [10]. Effective One Health interventions such as the improvement of the current prophylaxis in both humans [11,12] and dogs should enable reaching this goal.

Despite valuable efforts in several endemic countries [9,13,14], control strategies have not stopped rabies from circulating due to inadequate political, economic, and social responses. Weak interest from veterinary services, lack of sustainable resources and political neglect [15] prevent most endemic countries to reach the 70% vaccination coverage recommended by the WHO[9]. Moreover, rabies infections continue to spread, notably in previously rabies-free areas in countries such as Indonesia [16–18] and the Philippines [19,20]. In this resource-limited context, in-depth knowledge of the mechanisms underlying rabies dynamics (environmental drivers of spread, impact of dog density, impact of dog behavior, etc.) would be a key asset to limiting the spread of this vaccine-preventable disease, notably by aiding to design more effective vaccination campaigns that are robust to resurgence in the long-term. The development of novel methodologies to better understand rabies epidemiology and transmission dynamics therefore constitutes a promising avenue of research.

### Objectives

In this scoping review, we focused on the insights of two quantitative approaches applied to the study of rabies: mathematical modelling of infectious diseases and phylodynamics. The former is a field of research that exploits epidemiological data to unravel the spread of diseases in populations, assess the impact of interventions, support policy making, and optimize control strategies. The latter studies the interactions between epidemiological, immunological, and evolutionary processes from the analysis of viral genetic sequence data [21]. Within phylodynamics, phylogeographic inference specifically aims at reconstructing the dispersal history and dynamics of viral lineages in space and time. Here, we assessed the uses and respective contributions of both approaches, as well as their limitations and the remaining knowledge gaps concerning rabies dispersal and control in domestic dog populations.

## Methods

### Search strategy

**Fig 1:**
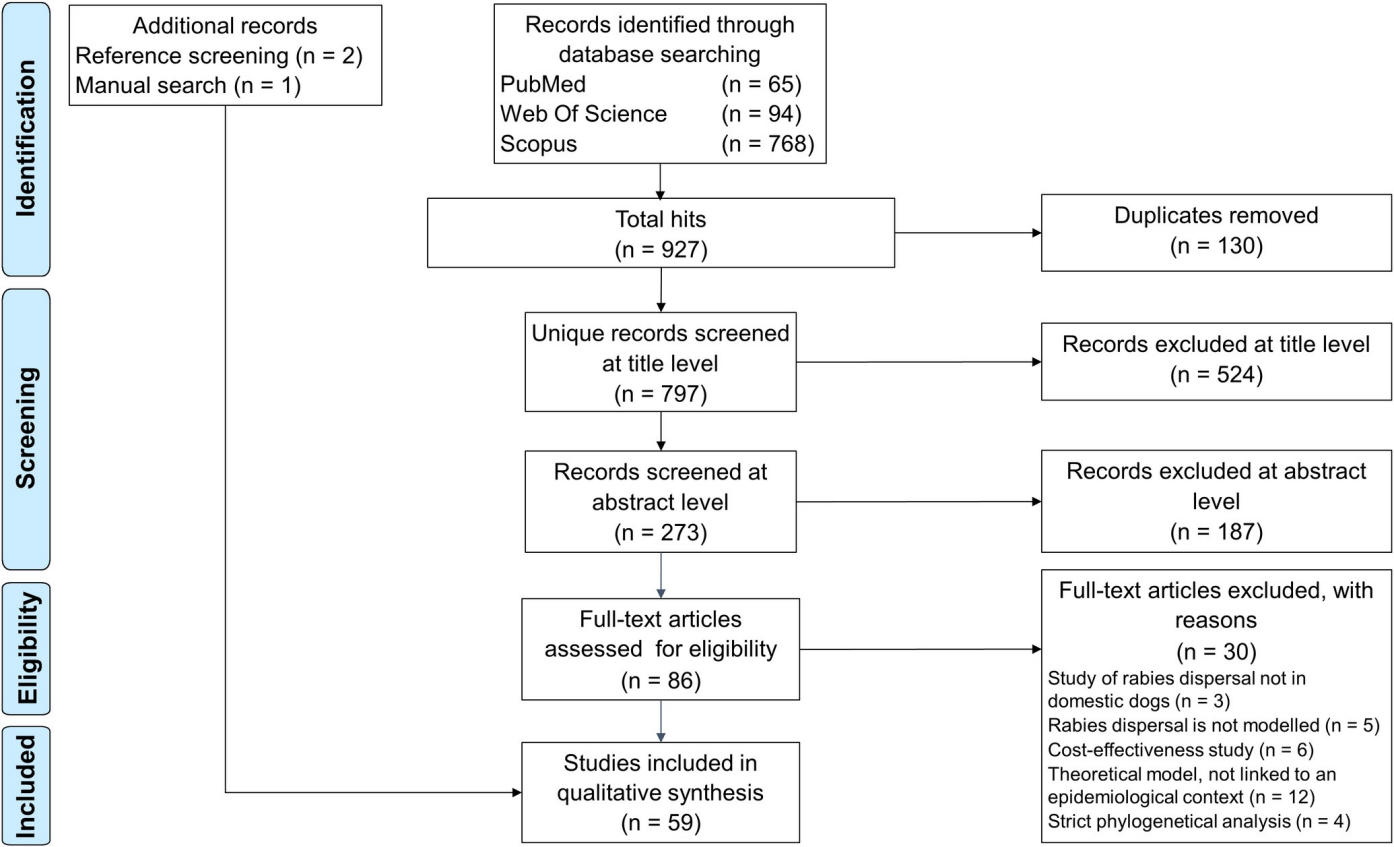
PRISMA-ScR Flow Diagram showing the number of identified and selected records along the multi-stage selection process. Scopus accounted for most of the records as it retrieved 71% (*n* = 46) of PubMed records and 79% (*n* = 74) of Web of Science records.

This review follows the guidelines of the PRISMA-ScR (Preferred Reporting Items for Systematic Reviews and Meta-Analyses Extension for Scoping Reviews) statement for scoping reviews [22]. In this review, we screened PubMed, Web of Science and Scopus databases on the 2nd of June, 2020 using the following combination of terms [“rabies” AND (“dog” OR “canine”) AND (“modelling” OR “modeling” OR “phylogeography” OR “phylodynamics”) AND “dynamics”] along with the “all fields” option and without restriction on publication year. The “all fields” option enabled to apply the search terms for their appearance in the title, abstract and keywords. Only English-written papers published in scientific journals were considered. All data were searched and screened by the same researcher (ML). The search strategy identified 65, 94 and 768 publications in PubMed, Web of Science and Scopus databases respectively, which corresponded to 797 unique records. In addition, references of selected publications were screened manually, leading to the identification and inclusion of two additional studies [23,24]. Finally, the paper of Colombi et al. [25], which was not identified in the databases nor in the references, was also included (Fig 1).

### Selection of studies

In total, 797 records were included and processed manually in a multi-stage procedure. At each selection step, a conservative approach was taken to ensure the best sensitivity level. Firstly, studies were selected based on their title using the following inclusion criteria: mathematical models of dog and human rabies assessing the impact of control strategies, the risk of rabies importation, the drivers of rabies spread or models estimating epidemiological parameters, cost-effectiveness studies, phylodynamic studies including RABV isolated from dogs, and broad studies on new phylodynamic or mathematical models. Indeed, rabies has often been used as a model disease in phylodynamics and mathematical modelling, and a reference to rabies might not appear directly in the title or the abstract. The following exclusion criteria were used: reviews, studies strictly on wildlife rabies, dog ecology and population dynamics, conservation biology, and evolutionary analyses for diagnostic purposes. Secondly, studies were selected based on their abstract with a refined set of exclusion criteria to exclude statistical analyses of epidemiological data, cost-effectiveness studies with no focus on rabies dynamics, experimental rabies cross-species transmission which did not incorporate a modelling aspect and studies on the evolutionary processes of RABV. Finally, studies went through a full-text reading step to verify that their content matched our selection criteria. At this step, theoretical models which were not grounded in a specific epidemiological context were excluded (Fig 1).

### Data extraction and analysis

Selected studies were classified into three categories based on their methodology: mathematical models, phylodynamic and interdisciplinary studies. Most phylodynamic studies identified in this review correspond to phylogeographic analyses, where the main focus is on inferring the spread of a pathogen over time using location data associated with the available sequence data. The interdisciplinary category covers studies either integrating epidemiological and genetic data in a unified modelling framework or mixing modelling approaches with phylodynamics. Data were systematically charted in an Excel spreadsheet designed to retrieve: *i*) the main modelling strategy with its assumptions; *ii*) the data source; *iii)* remarks about potential bias of the data in relation to the underlying evolutionary and epidemiological processes; *iv*) the qualitative and quantitative results concerning the dynamics of dog rabies; and *v*) if performed, the sensitivity analysis determining the robustness of the methodology to parameter values or potential biases.

## Results

### General characteristics of selected studies

**Fig 2:**
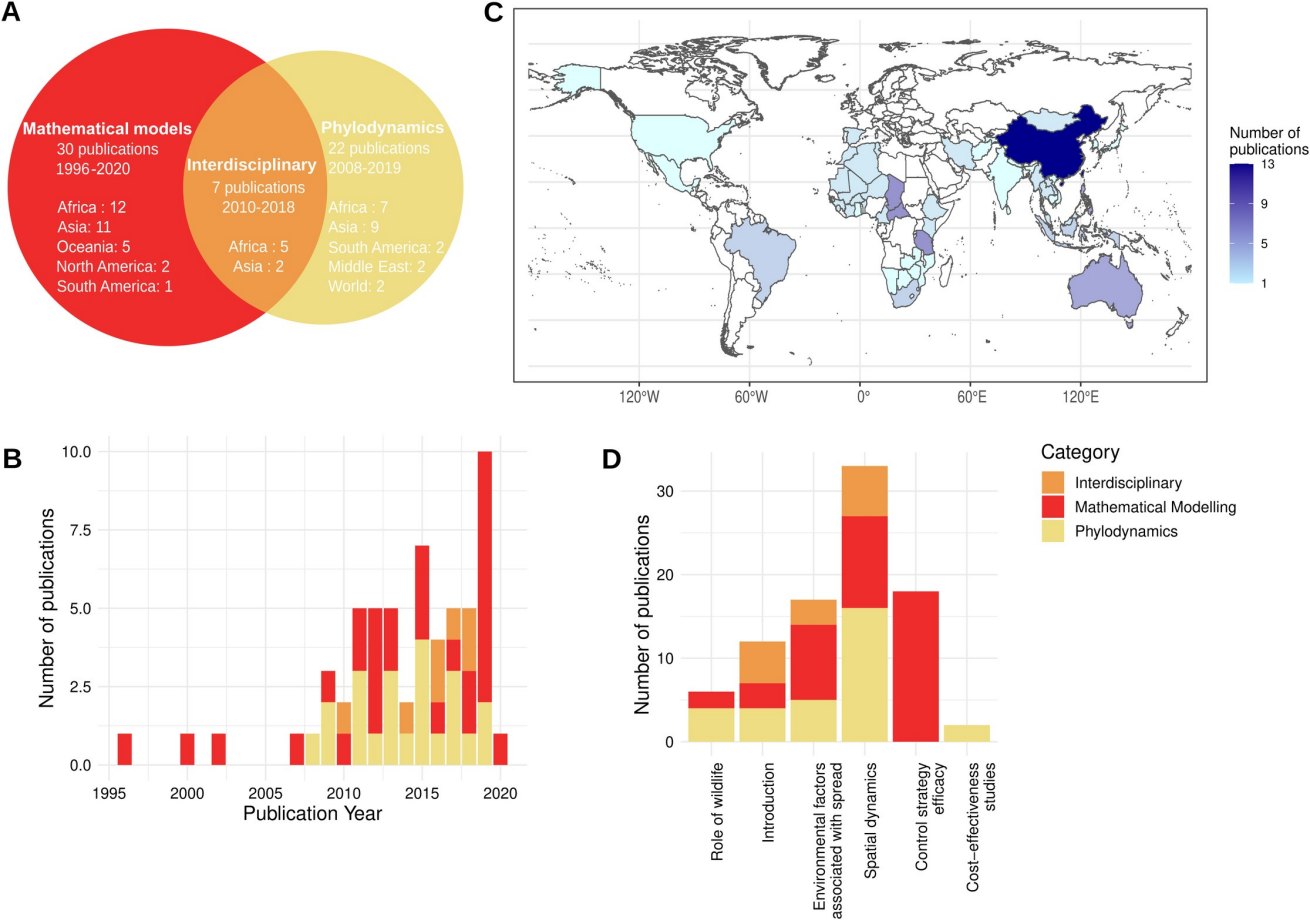
General characteristics of the selected dog rabies studies. (A) Classification of the included publications with the total number of studies, the publication time span, and the number of publications per continent of study. Asia and Africa account for up to 78% of the included studies. (B) Number of publications per year and per methodological category. Mathematical models were the first studies to be published followed by phylodynamic and interdisciplinary studies. (C) Number of publications per country of study. Each publication was attributed to one or multiple countries based on the origin of the RABV genetic sequences, rabid case data or dog ecology data. For phylodynamic studies, countries were not considered if their genetic data were included only in regular phylogenetic tree reconstructions. Similarly, two studies which described rabies dynamics at the global scale [52,65] were not considered in this figure. In our collected records, China accounts for most Asian studies. Spain appears on the map because Ceuta and Melilla, which are Spanish enclaves in North Africa, are represented in two datasets of RABV genetic sequences [68,72]. (D) Number of studies per topic and methodological category. The World Bank, https://datacatalog.worldbank.org/dataset/world-bank-official-boundaries, CC-BY 4.0.

Our selection procedure identified 59 studies that meet our selection criteria with 30 mathematical models [16,23–51], 22 phylodynamic studies [17,19,52–71], and 7 interdisciplinary studies [20,72–77], all published between 1996 and 2020 (Figs 1 and 2A and 2B). Mathematical models were first published followed by phylodynamic and interdisciplinary studies (Fig 2B). This timeline can be explained by the recent developments of Bayesian phylodynamic, and in particular phylogeographic, models in BEAST [78–80]. Africa and Asia are the most studied continents in the three methodological categories, while China accounts for most of the Asian studies (Fig 2C). Oceania is not represented in the interdisciplinary and phylodynamic categories since it is a rabies-free area (Fig 2A).

### Topics addressed by the studies

Phylodynamic studies are homogeneous in terms of methodologies (essentially phylogeographic studies) and research goals. They predominantly focus on unraveling the dispersal dynamics of rabies at the regional and country levels (*n* = 16) [17,19,52–58,61,63,64,67,68,70,71]. In four of them, the authors deciphered the role of lineage introduction in rabies maintenance or emergence [59,60,62,66]. In recent years, researchers have been trying to identify external factors impacting the spatial dynamics of RABV spread (*n* = 5) [63,64,68,69,71] (Fig 2D and S1 Table). Contrary to phylodynamic studies, the modelling category gathers a diverse panel of models with aims that cover the implementation of new mathematical methodologies (*n* = 2) [42,46], the characterization of rabies dynamics (*n* = 11) [26,27,31,32,40,41,44,47–49,51], the identification of factors driving the resurgence or maintenance of rabies (*n* = 9) [16,23,25,33–35,37,38,43], the assessment of control strategies efficacy (*n* = 18) [16,23,24,27–29,31,33–36,42–45,49–51], the risk assessment of rabies introduction and the evaluation of outbreak preparedness in rabies-free areas (*n* = 3) [30,36,42], and cost-effectiveness studies (*n* = 2) [39,48] (Fig 2D and S1 Table). Finally, interdisciplinary studies mainly focused on rabies dynamics in endemic areas (*n* = 6) [20,72–74,76,77] and the identification of environmental factors influencing rabies spread and maintenance such as recurrent reintroductions (*n* = 3) [72,75,77]. Two of these used genetic and epidemiological data of dog rabies in a unified modelling approach [73,76], whereas the others analyzed sequences through regular phylogenetic approaches and completed their analysis with a mathematical model [20,72,74,75,77] (Fig 2D and S1 Table).

### Potential sources of bias in the data

Data source (active/passive surveillance), resolution (number and length of RABV sequences, incidence per country/region, etc.) and representativity influence the level of evidence of the studies on the underlying epidemiological and evolutionary processes. In particular, recorded cases collected through passive surveillance systems are expected to underestimate the disease burden and to be potentially spatiotemporally biased [8,81]. Similarly, genetic sequences collected from publicly available databases such as GenBank often lack precise metadata (e.g., sampling time and location) and/or are of short length.

In our text corpus of phylodynamic and interdisciplinary studies, passive surveillance systems and GenBank represent the main sources of RABV genetic sequence data (S2–S4 Tables). By combining these two data sources, researchers have generally managed to increase the spatiotemporal coverage of their dataset. This however does not guarantee a good representativity of the epidemic process. Active surveillance was mostly used to collect dog specimens from animal markets in China (*n* = 2) [58,60] and thorough contact tracing after biting events in China and Tanzania (*n* = 2) [63,66]. On average, the datasets analyzed in these studies contained 183 sequences spanning from approximatively 3% to 100% of the RABV genome length. Short sequences containing the N gene constitute the most common type of data. They are less informative than whole genomes which were only generated and analyzed in recent years across four studies [63,65,69,71] (S2 Table).

In studies from the modelling and interdisciplinary categories, authors generally simulated rabies epidemics (*n* = 24) [20,23,25,28–36,38,40,42–47,49–51,72], and thus predominantly relied on publicly available estimates of the natural history of rabies, dog demographics and dog ecology (S3 and S4 Tables). When models were fitted to incidence data (*n* = 13) [16,24,26,27,37,39,41,48,73–77], human and/or dog case data from passive surveillance systems were used, or bite incidence data from thorough active surveillance. In general, there was a lack of data on dog rabies cases (available in 10 studies; [16,24,26,27,37,48,73–77]) and estimates on dog demographics and ecology integrating the local specificities of host ecology were available in only seven studies [27,37,39,41,48,75,77]. Access to local data is crucial since differences in rabies spread [27] and dog carrying capacities [39] were estimated between areas of the same country. We would expect these differences to be more pronounced across different countries. To overcome the lack of epidemiological data on dog rabies, one study used serological data (from vaccination campaigns) to model the dynamics of rabies [46], and another study [36] based its analyses on historical records in Japan from the 1950s. Similarly, most Australian studies [30,42–44] took the perspective of dog ecology data since Australia is free of rabies. This way, the authors explored the impact of dog population structure and dog roaming behavior on rabies dynamics.

### Description of the models

In studies using phylodynamic approaches, the geographical dispersal of rabies was studied using either parsimony (*n* = 4) [52,54,55,58], Bayesian discrete phylogeography (*n* = 18) [17,19,20,53,56,57,59,60,62–64,66,67,70–72,77,82], or Bayesian continuous phylogeography (*n* = 6) [61,68,69,71,74,77] (S2–S4 Tables). All Bayesian phylogeographic studies were carried out in BEAST 1 [79] with discrete trait analysis (DTA) to perform a phylogeographic reconstruction based on discrete/discretized sampling locations (e.g. provinces or countries) or with continuous trait analysis to perform a phylogeographic reconstruction based on spatially-explicit sampling location data (latitude and longitude coordinates). Several methodologies take advantage of such phylogeographic inferences to investigate the impact of external factors on the dispersal of viruses: a generalized linear model (GLM) extension of DTA developed by Lemey et al. [83] to test predictors of dispersal transition frequencies among discrete locations which was implemented by Brunker et al. [69]; and *post hoc* statistical approaches developed by Dellicour et al. [71,84,85] to investigate the impact of environmental factors on the dispersal velocity, direction, or frequency of viral lineages in continuous phylogeographic frameworks which were applied in four rabies studies [68,69,71,77]. Finally, Zinsstag et al. [75] were the only authors to implement a birth-death model in BEAST 2 [80] to reconstruct the effective reproduction ratio (R) along vaccination campaigns and compare it to estimates obtained with a modelling approach (S4 Table).

Compared to phylodynamics, mathematical models display a large diversity of specifications and parametrizations. Compartmental models (*n* = 18) [20,23,24,26,27,33,34,39–41,45–49,51,75,77] are the most represented models, followed by agent-based (*n* = 8) [16,30,31,35,36,42–44] and metapopulation (*n* = 5) [25,28,32,37,50] models. Other model types such as network models or branching processes are also represented [29,38,73,74,76] (S3 and S4 Tables). The development of new dog rabies models builds upon the literature since 15 models out of the 37 identified were adapted from previously published dog rabies or wildlife rabies models (S3 and S4 Tables). This is the case notably for compartmental models which correspond to the simplest models of rabies dynamics. Metapopulation, agent-based, and other model types are more complex, in that these approaches often integrate spatial dynamics of dog rabies [25,30,32,35–38,42,43,72,73,76].

Population structure can be integrated in any modelling framework under the form of contact heterogeneity, age-structured populations, roaming behavior, or individual heterogeneity. In compartmental models, population structure is integrated either as a set of strata (stray dogs, owned free-roaming dogs, owned confined dogs) interacting together [33], or by specifying a structured next-generation matrix from which R is generally derived [34]. Such models are also referred to as multi-host models and may integrate other hosts: humans [32,39,40,48,49,51,86], cattle [39], wildlife [27,41]. In agent-based and network models, population structure is defined at the individual level using spatial kernels [16,25,30,31,36,42], individual contact rates [30,35,44], vaccination status [30,36], life span, infectious period [16,31,44], etc.

### Sensitivity analyses

Sensitivity analyses are commonly used to assess the robustness of inference to both data representativity and model specifications, and to identify the most influential parameters on specific model outputs. In our text corpus, no sensitivity analyses were found to be carried out in phylodynamic studies which can be attributed to the relatively small number of sequences analyzed in those studies. In contrast, sensitivity analyses were commonly performed in mathematical models, either to unravel the key parameters influencing rabies dynamics or to verify the robustness of the results to model assumptions. Dog ecology parameters such as birth rate and carrying capacities are often reported as key parameters on rabies dynamics predictions although they are not estimated using local data. Transmission rates are also determinant in model predictions (S3 Table). In spatially explicit studies, mobility parameters also have a strong impact on model inferences. Finally, the impact of under-reporting was tested only in interdisciplinary studies, two of which reported a strong impact of the reporting rate on model inference [20,76] whereas the other two were robust to a change in this parameter [74,75] (S4 Table).

### Insights into dog rabies dynamics and its drivers from phylodynamic and modelling studies

Phylogeographic analyses have aimed to unravel the spatial dynamics of dog rabies at the global and regional scales and showed that dog RABV lineages cluster spatially at the global scale, except for one lineage, referred to as the cosmopolitan lineage, which is largely distributed across the world [52]. At the regional and country scales, there is co-circulation of dog-related lineages, notably in China [55,58,64,66,70], in the Middle East [62,71], as well as in Western and Central Africa [54]. However, each lineage exhibits a strong geographical structure. In the case of country-specific lineages, various studies have suggested that transboundary movements are not a major force of rabies dispersal [19,53,54,59,60,68]. All study categories unraveled the role of human-mediated movements in rabies spread. Overall, phylogeographic analyses provided evidence for the effect of anthropogenic factors: major roads are associated with rabies dispersal in North Africa [72], and RABV lineages tended to preferentially circulate within populated areas in North Africa [68] and the Middle East [71]. Other factors are associated with rabies spread in Yunnan (China, Tables 1 and S5). These results may reflect the intimate link between rabies dynamics, host ecology and dog-human interactions. Mathematical models highlighted the short length of canine rabies transmission chains [31,73,76] and unraveled the importance of long-range human movements in disease spread [25,32]. Finally, interdisciplinary approaches highlighted the crucial role of long-distance transmission events likely due to humans in rabies dynamics in North Africa [72] and also showed that main roads act as barriers to dog rabies dispersal in an urban setting in Africa [35].

**Table 1:** Estimated parameters in phylodynamic models.

Location	Sampling window	Viral lineages	RABV sequence	Migration rate (migrations.year^-1^, 95% HPD)	Velocity^a^ (km.year^-1^, 95% HPD)	Diffusion coefficient (km^2^.year^-1^, 95% HPD)	Factors facilitating viral spread^b^	Factors impeding viral spread^b^	Reference
Bangui, Central African Republic	1986–2012	Africa 1 Africa 2	N P M G intergenic G-L	-	v = 0.9 (0.65–1.2)	-	-	-	Bourhy et al., 2016 [74]
Serengeti district, Tanzania	2004–2013	Africa 1b	Whole-genome	-	v = 4.46 (3.22–5.88) Coefficient of variation M = 3.10	-	Dog presence	Elevation Rivers	Brunker et al., 2018 [69]
Yunnan province, China	2008–2015	SEA-1 SEA-2 SEA-3	N G	-	v = 57.5 (39.2–85.1) v_weighted_ = 23.4 (2.4–32.6)	D = 1733 (1082–2928) D_weighted_ = 1064 (116–1638)	Forest coverage (but with a tendency to spread towards areas associated with relatively low forest coverage)	-	Tian et al., 2018 [77]
North and Northeast regions, Brazil	2002–2005	-	N	-	v_overall_ = 12.88^c^ v_dogs_ = 30.5^c^ v_*cerdocyon thous*_ = 9.0^c^	-	-	-	Carnieli et al., 2013 [61]
Algeria	2001–2008	Africa 1	N P intergenic G-L	-	v_great circle distances_ = 26 (18–34) v_road distances_ = 33 (23–43)	-	Major roads	-	Talbi et al., 2010 [72]
Algeria	2001–2008	Africa 1	N P intergenic G-L	-	v_wavefront_ ~ 15^c^	D = 2874 (1900–5420) D_weighted_ = 1305 (1086–1574)	Grasslands Urban areas	Elevation	Dellicour et al., 2017 [68]
Morocco	2004–2008	Africa 1	N P intergenic G-L	-	v_great circle distances_ = 42 (26–58) v_road distances_ = 51 (34–72)	-	Major roads	-	Talbi et al., 2010 [72]
Morocco	2004–2008	Africa 1	N P intergenic G-L	-	v_wavefront_ ~ 22^c^	D = 2874 (1900–5420) D_weighted_ = 1305 (1086–1574)	Grasslands Urban areas	Elevation	Dellicour et al., 2017 [68]
Iran	2008–2015	-	Whole-genome	-	v = 55.5 (38.9–142.4) v_weighted_ = 18.1 (16.3–20.8)	D = 2676 (1935–5066) D_weighted_ = 1643 (1356–2325)	(Tendency to spread towards and preferentially circulate within accessible areas associated with relatively higher human population density)	(Tendency to avoid circulating in barren vegetation areas and to avoid spreading towards grasslands)	Dellicour et al., 2019 [71]
China	1983–2016	Arctic-like 2 Central Asian 1 SEA-1 SEA-2 SEA-3 SEA-5	N	5.81e-3 (3.92e-3–7.77e-3)	-	-	-	-	Wang et al., 2019 [70]

Abbreviations: HPD, Highest Posterior Density; SEA-1, South-East Asia 1; SEA-2, South-East Asia 2; SEA-3, South-East Asia 3.

The sampling window and the spatial scale of the studies are highly variable. Thus, it is not possible to directly compare the velocity and diffusion coefficients amongst the different study settings.

a Depending on the study, estimates of RABV lineage velocity or diffusivity were obtained by estimating different dispersal statistics. Talbi et al. [72] reconstructed for each branch of the phylogenetic tree the expected number of migrations between two locations using a discrete phylogeographic model. The authors multiplied these estimates by the great-circle distance between the two locations, and thus, obtained the expected distance travelled within the time elapsed on each branch. Carnieli et al. [61], Bourhy et al. [74], Brunker et al. [68], Tian et al. [77], and Dellicour et al. [70] estimated the mean branch velocity using continuous phylogeographic reconstructions. Finally, Dellicour et al. [67] estimated the temporal evolution of the wavefront velocity that corresponds to the distance between the reconstructed epidemic origin and the maximal epidemic wavefront. While the mean branch velocity (v) and diffusion coefficient (D) are estimates of the dispersal velocity and of the diffusion coefficient averaged over all tree branches, respectively, their weighted average counterparts involve a weighting by branch time resulting in lower-variance estimates [70].

b Depending on the study, the impact of environmental factors on dispersal of viral lineages were investigated using different approaches. Talbi et al. [72] simulated random or conditional dispersal of RABV in northern Africa along phylogenetic trees reconstructed by phylogeographic inference and compared simulated dispersal patterns with the observed spread. Brunker et al. [68] parametrized a generalized linear model (GLM) in a discrete phylogeographic framework with resistance distances derived from landscape data between clusters of rabies cases. Dellicour et al. [67] and Tian et al. [77] assessed which environmental factors are associated with RABV velocity using continuous phylogeographic inference and post hoc statistical analyses. Dellicour et al. [70] and Tian et al. [77] also identified factors associated with the direction of spread using phylogeographic reconstructions and subsequent post hoc analyses.

c 95% Highest Posterior Density (HPD) intervals are not specified in the original publications.

Phylodynamic studies showed that introduction through infected dog movement is the major force of rabies spread towards disease-free areas, as Indonesia [16–18] and the Philippines [19,20] have recently experienced, and also represents a driver of rabies spread in endemic areas where frequent reintroductions counteract local rabies elimination after vaccination campaigns [74,75]. In these settings, phylodynamic analysis constitutes a powerful tool to confirm introduction events [19,56,59,72,74,75]. Multiple mathematical models have also shown that frequent reintroductions drive rabies persistence in endemic areas [31,37,73,76].

Population structure constitutes another driving force of rabies maintenance as explored in simulation studies integrating dog ecology data in Australian [30,42–44], Japanese [36], Tanzania [28,50] and Chadian [35] settings. Rabies-induced behavioral changes were shown to contribute to rabies persistence in small dog populations [44] as well as differential roaming behavior, contact rates between dog strata and the structure of contact networks [30,34–36,44].

The contribution of wildlife to canine rabies spread and maintenance is rarely addressed in phylodynamic studies because viruses isolated from wildlife specimens often correspond to dog-related lineages [19,56,64,66,70] or because of insufficient sampling efforts when it comes to wildlife [58] (S1 Table). Nevertheless, specific RABV lineages were shown to circulate both in wildlife and domestic dogs in the Middle East and Tanzania with complex interspecies transmissions [62,65,69,71]. A phylodynamic study at the global scale showed that host shifts from dogs to wildlife with adaptation to the new host were common in RABV history [65], which may explain why different lineages circulate in dogs and wild foxes in Brazil [61], in dogs and ferret badgers in Asia [65] and in dogs and mongooses in South Africa [65] with rare interspecies transmission events. By incorporating direct interspecies transmission, mathematical modeling studies showed that dog population contributes to sustained rabies circulation in wildlife instead of the other way around [27,41]. Similarly, the proximity to wildlife was shown to not impact rabies spread in dogs in the model of Beyer et al. [28].

**Fig 3:**
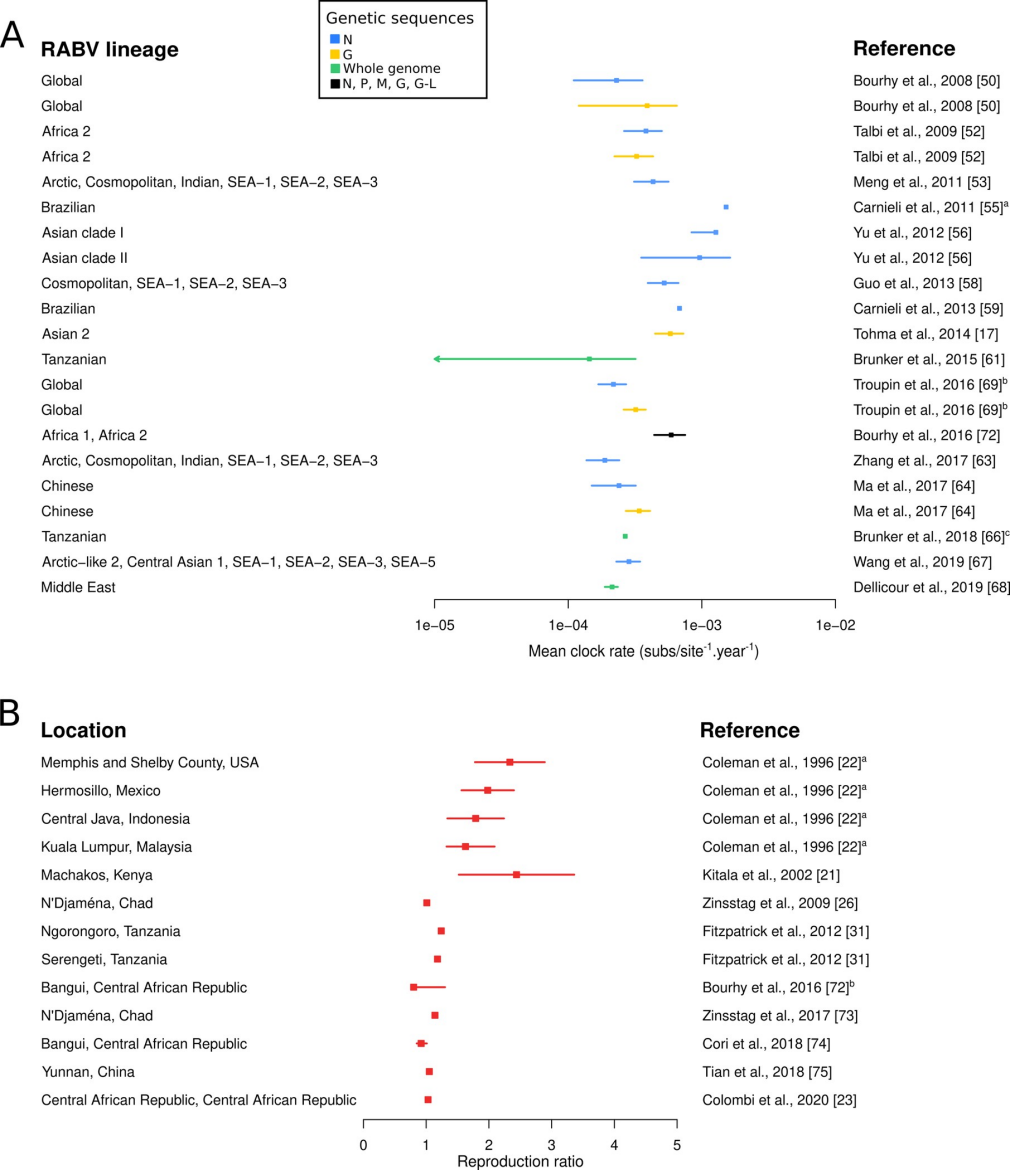
Estimates of the mean evolutionary rate of RABV and the reproduction ratio of canine rabies in the included studies. (A) Bayesian credibility intervals (mean and 95% Highest Posterior Density, HPD) of the mean evolutionary rate of canine RABV per genetic sequence and RABV lineage. ^a^The estimate corresponds to the upper bound of the 95% HPD. ^b^The dot corresponds to the median and the interval to the 95% HPD interval. cThe 95% HPD was not specified in the original publication. (B) Estimates of the reproduction ratio of dog rabies per control strategy or geographical location. The dot corresponds to the mean and the interval to the 95% confidence interval unless stated otherwise. ^a^ The interval corresponds to the standard error. ^b^ The authors estimated the effective reproduction ratio along time. Here, the value range of the median monthly point estimate is depicted.

Finally, mathematical models and phylodynamics provide convenient estimates of a range of parameters on rabies dispersal dynamics (lineage dispersal velocities, diffusion coefficients; Table 1), rabies evolutionary processes and dog ecology. For example, the evolutionary rate was homogeneously estimated to be between 1 x 10^−4^ and 5 x 10^−4^ substitutions per site per year across RABV genes and lineages, except for the Asian lineage which is estimated to evolve faster (Fig 3A). The time to the most recent common ancestor (TMRCA) is also frequently estimated in phylodynamic studies (S2 Table) which is generally more recent than suggested by historical records. R, the expected number of secondary infections, is often estimated by fitting case data to mathematical models (Fig 3B) or by computing its value based on the choice of parameters value (S6 Table). Its estimate ranges from 0.80 to 3.36 according to the setting but it is generally estimated to be between 1 and 2, corresponding to a low-grade transmission with frequent stochastic extinctions. Other parameters such as the dog-to-dog transmission rate, the introduction rate or the dog carrying capacity are also frequently estimated (S6 Table).

### Effective control strategies

Interdisciplinary and modelling studies generally assessed the impact of past or potential control strategies to eliminate dog rabies. The specifications of the explored control strategies depended on the economic situation of the country in which the study was supposed to be performed, as well as the model type. Dog vaccination was the most studied control measure (*n* = 28) [16,23,24,26–28,30–37,39–41,43–51,75,77], whereas culling (*n* = 7) [30,33,42,45,48,49,51], dog confinement or movement ban (*n* = 4) [30,31,36,42], control of dog birth rate (*n* = 4) [40,45,49,86] and community behavior (*n* = 1) [31] were rarely modelled. Culling was shown to be effective in two compartmental model studies [45,51] while vaccination was generally found to be the most effective strategy. Vaccination coverage strongly depends on the setting: 90% or complete dog vaccination coverages are recommended in rabies-free areas with high surveillance and control capacities whereas lower coverages associated with complementary strategies are recommended in endemic areas (Table 2). Nevertheless, the efficacy of vaccination strategies is mitigated by new introductions due to neighboring transmission or long-distance movements mediated by humans [25,29,31,37,74,75,87], notably in low vaccinated populations [32]. In this case, reactive vaccination strategies [16] or dog movement bans [25] constitute alternative effective measures. However, Ferguson et al. [31] evaluated the impact of new introductions in vaccinated areas, and concluded that vaccination coverages were robust to rabies introduction in their specific setting. Similarly, Beyer et al. [50] suggested that the spatial structure of dog population had more impact than rabies introduction on the efficacy of vaccination campaigns. In terms of vaccination coverage, successful vaccination campaigns should target homogenous coverage since hidden pockets of rabies transmission might jeopardize control efforts [16,23,29,31]. In terms of campaign frequency, the efficacy of pluriannual compared to annual vaccination campaigns is difficult to evaluate as it results from many factors including the number of vaccination pulses, the time interval between each pulse, dog birth rate and the introduction rate of infectious animals [23,28,45].

**Table 2:** Recommended control strategies in mathematical modelling studies.

Epidemiological context	Recommended control strategy	Specificities of the recommended control strategie	Location	Reference
Introduction in previously rabies-free areas	Reactive dog vaccination	Followed by a 2-year monitoring period		Townsend et al., 2013 [29]
		Until all targeted dogs are vaccinated	Northern Peninsula Area and Elcho Island, Australia	Dürr et al., 2015 [30]
	90% dog vaccination coverage		Northern Australia and New South Wales, Australia	Sparkes et al., 2016 [33]
			Kubin, Saibai and Warraber divisions, Australia	Brookes et al., 2019 [44]
	Targeted dog vaccination campaigns	Vaccination of free-roaming dogs	Northern Peninsula Area, Australia	Hudson et al., 2019 [43]
	Integrated approach	Mandatory dog vaccination Dog owner awareness Dog registration, Capture of free-roaming dogs Quarantine of imported animals	Ibaraki and Hokkaido prefectures, Japan	Kadowaki et al., 2018 [36]
Endemic areas	90% dog vaccination coverage		Lemuna-bilbilo and bishoftu districts, Ethiopia	Beyene et al., 2019 [39]
	75% dog vaccination coverage	Stray dog management	Guangdong, China	Hou et al., 2012 [51]
	70% dog vaccination coverage	Annual vaccination (or biannual vaccination with a 60% coverage)	Machakos district, Kenya	Kitala et al., 2002 [23]
			N’Djaména, Chad	Zinsstag et al., 2009 [48]
		Even coverage	Bali, Indonesia	Townsend et al., 2013 [16]
			Serengeti and Ngorongoro districts, Tanzania	Fitzpatrick et al., 2012 [27]
	≥50% dog vaccination coverage	≥ 50% fertility control coverage		Carroll et al., 2010 [45]
			Sarawak state, Malaysia	Taib et al., 2019 [40]
	Even dog vaccination coverage		Region IV, Philippines	Ferguson et al., 2015 [31]
	Targeted dog vaccination campaigns	Frequent dog vaccination campaigns targeting the reduction in metapopulation risk	Serengeti district, Tanzania	Beyer et al., 2012 [28]
		Stray dog vaccination coverage based on dog population composition		Leung et al., 2017 [34]
		Vaccination based on social and roaming behaviors Public awareness Locally reactive interventions Reporting of 60% of cases by the surveillance system	N’Djaména, Chad	Laager et al., 2018 [35]
	Dog population management	Dog vaccination Public awareness	China	Zhang et al., 2012 [26]
		Massive dog vaccination campaigns in urban areas Dog movement bans	Central African Republic	Colombi et al., 2020 [25]
		Dog vaccination	China	Zhang et al., 2011 [49]

The efficacy of control strategies on dog rabies dynamics has been addressed in only a subset of the currently available mathematical modelling studies. Studies presented in this table compared several control strategies or different dog vaccination coverages on rabies elimination prospects. The optimal control strategy inherently depends on the epidemiological context (endemic or introduction in previously rabies-free areas), the setting (local surveillance and vaccination capacities), the assumptions of the dog rabies model and the control strategies tested by the researchers. Here, we report the strategies recommended by the authors which include quantitative and qualitative criteria such as the estimated impact of public awareness on rabid dog detection and management. Three studies [35,40,51] are not grounded in a specific geographical area. Using simulated scenarios, they test the impact of control strategies according to the time to detection [35], dog population structure [40] and the use of immunocontraceptives [51].

Recent studies [28,34–36,42,43] proposed targeting at-risk dog populations, such as explorers and roaming dogs, to improve the efficacy of vaccination campaigns (Table 2). However, the sensitivity analysis of Laager et al. [37] showed that population structure did not impact the efficacy of vaccination strategies. There are conflicting results concerning stray dog vaccination which was either less efficient than owned dog vaccination [51] or dependent on population composition [34].

Several studies also suggested an impact of dog birth rate reduction on the incidence of rabies [23,26,40,41,45,49]. However, the cost and feasibility of dog population management strategies such as sterilization render this unfeasible in many settings [88]. Dog confinement, which is generally spontaneously put in place by local communities during rabies outbreaks, may improve elimination prospects but, when implemented, the level of confinement is not sufficient to reach elimination [25,30,31]. Concerning the rabies burden in humans, some studies recalled the importance of public awareness (Table 2) and proper PEP coverage to reduce the number of human cases, even though it does not impact rabies circulation in dogs [26,35,36,41]. All these findings confirmed and justified the strategic plan that provides a phased, all-inclusive, intersectoral approach to eliminate human deaths from rabies recently launched by United Against Rabies, in a collaboration between four partners: WHO, FAO, OIE and GARC [13].

## Discussion

### Insights on rabies epidemiology and control

**Fig 4:**
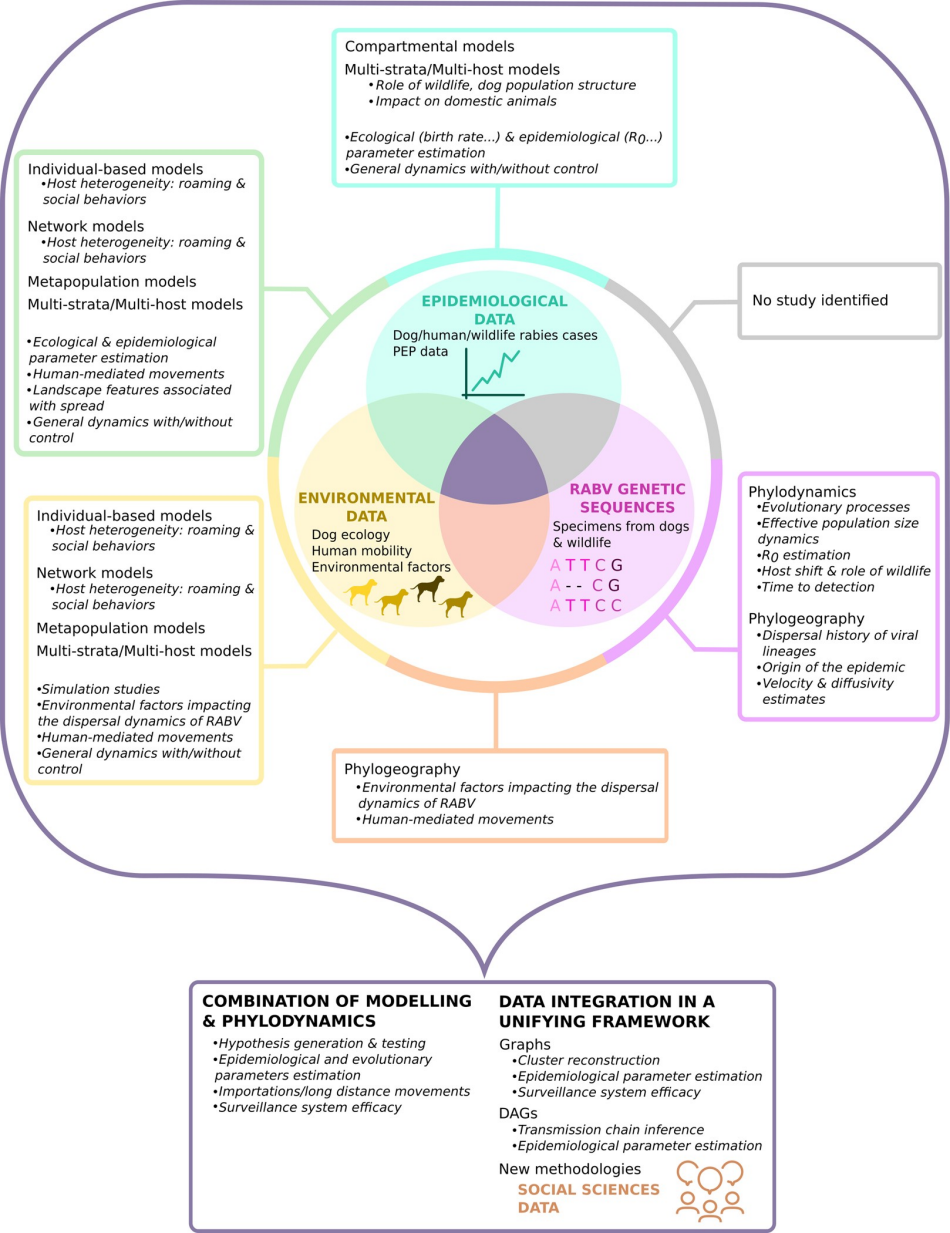
Visual summary of the uses of epidemiological data, environmental data and RABV genetic sequences for the study of rabies dynamics and control. Epidemiological data, environmental data, RABV genetic sequences and social sciences data are highlighted in cyan, yellow, pink, and brown, respectively. The section corresponding to models combining epidemiological data and RABV genetic sequences only is colored in grey since no study that meets this criterion has been identified using our search strategy. Models and their contributions to the understanding of rabies spread and control are detailed in the colored tags. Models using multiple types of data are colored with the intersection color of the corresponding data types. In our text corpus, few studies combined epidemiological, ecological, and genetic data in a unified framework.

In this review, we assessed the respective contributions of mathematical modelling and phylodynamics to the understanding of rabies spread and control in dog populations. Contrary to phylodynamic studies, mathematical modelling approaches were multi-faceted and mainly addressed the efficacy of control strategies and, less frequently, the drivers of rabies spread. They revealed the crucial role of frequent introductions and the potential role of dog population structure in disease dispersal and maintenance, as well as the overwhelming efficacy of dog vaccination campaigns over other control strategies. Certain studies also estimated key parameters of rabies dynamics and dog ecology, such as dog birth rate or dog carrying capacity. On the other hand, phylodynamic studies mostly focused on the description of viral dynamics at the global, regional, and local scales, and recently tested which environmental factors are impacting RABV spread. These approaches consistently unraveled the occurrence of long-distance movements suspected to be human-mediated and confirmed the role of humans in rabies dispersal dynamics in Africa and the Middle East. A third kind of studies either combined phylodynamics and mathematical modelling or presented new models integrating epidemiological and genetic data. In the former approach, hypotheses on rabies spread were generated and tested in the same epidemiological context, and thus, confirmed the impact of introductions and human movements in a low-grade transmission process characterized by small clusters and frequent stochastic extinctions. The latter approaches aimed at reconstructing local transmission chains or clusters, opening new horizons on data integration and the study of rabies (Fig 4). Unfortunately, a large number of endemic countries is still not, or poorly studied. Data collection and/or model formulation are still needed in Russia, and most of Africa, and South-East Asia.

The limitations of our review should be acknowledged. In preliminary analyses, we noticed a high variability in record selection according to the combination of search terms, and certainly due to the ambiguous use of specific terms such as phylodynamics in the literature. Since the studies selected in this review are mainly in line with previous reviews [82,89,90], we argue that we retrieved a large part of the available studies on rabies dynamics and control.

### Open questions in rabies epidemiology and control

In this review, we summarized the findings of mathematical modelling and phylodynamics on the factors that impact rabies spread. Nevertheless, the full picture of rabies epidemiology remains unclear. First, the role of dog roaming behavior, and dog contact networks in dog rabies spread should be further investigated. In this review, we identified seven studies [33–35,38,42–44], all situated in Australia and Africa, showing that highly connected dogs or free roaming dogs participate in a large part in the spread of the disease. By specifically targeting this type of dogs, vaccination campaigns could be more effective according to Leung et al., 2017 [34], Laager et al., 2018 [35], and Hudson et al., 2019 [43]. Yet only one study combined contact data with epidemiological data [35]. The ecological and behavioral drivers of rabies circulation in domestic dogs are still not fully understood. If stray dogs do constitute a major driver of rabies dispersal, this could have direct implications on the field concerning stray dog population management for example.

Additionally, the role of wildlife and other host species remains unclear [91]. Even though the circulation of dog rabies seems predominant in dog populations, there are too few studies addressing the dynamics of RABV in wildlife and dogs. Furthermore, the interactions between dogs and other carnivore species are expected to change from location to location. Indeed, the interactions between dog populations and wild carnivores depend on the abundance of wild populations and the frequency of contacts between the dog reservoir and wildlife [27,41]. Better understanding the role of wildlife could also have direct implications on local policies such as increasing public awareness, notably in rural areas or strengthening wildlife surveillance systems for rabies.

At a broader scale, the spatial dynamics of rabies are still poorly understood. Urban areas were first thought to be hubs of rabies transmission but recent studies have shown that rabies could be eliminated temporarily at the city-level through mass dog vaccination campaigns [37,74,75]. These case studies suggest that urban areas are not hubs of rabies transmission but part of the complex spatial heterogeneity of dog ecology and dog movement. By exploring the dynamics of dog rabies circulation in urban, peri urban and rural areas, rabies research could see an improved understanding of rabies ecology. This could have direct implications on the design of vaccination campaigns, by prioritizing vaccination campaigns in hubs of rabies transmission, followed by locations with intermediate and low transmission.

Finally, there is extensive evidence of the efficacy of dog vaccination to control the spread of rabies in both human and dog populations. We showed in this review that higher coverages are recommended in rabies-free areas than in endemic areas, however, the practicalities of vaccination campaigns are rarely addressed. As a neglected tropical disease, rabies control programs are designed and deployed in resource-limited contexts. Thus, high, and even intermediate vaccination coverages cannot be achieved at once over a large area. The periodicity, the spatial prioritization, the targeted populations, and the association with other control strategies (dog population management, dog movement ban…) are interesting modalities that could be tested in models and could substantially improve resource allocation.

### Future directions of mathematical modelling and phylodynamics for rabies research

There is an evident lack of extensive and adequate databases possibly due to restricted data collection, data accessibility and/or data analysis capacity in resource-limited settings [92,93]. This constitutes the main weakness of mathematical modelling and phylodynamic studies that we identified in this review (Table 3). Epidemiological and ecological (census data, population structure, contact networks) data are needed to account for local specificities in terms of modeling interactions between rabies virus (RABV), dog reservoir, domestic animals, wildlife reservoir and human populations. Similarly, there is a need for longer RABV genetic sequences and more thorough sampling to discriminate fine-scale migration events and better characterize the interactions between RABV lineages [63,82,94]. Improving operational data collection is nevertheless challenging. Genomic surveillance relies on laboratory infrastructures, supply chains and expertise, all of which are costly and generally lacking in low- and middle-income countries. New portable sequencing technologies combined with bioinformatics workflows could accelerate capacity building through portability and affordability [94,95]. In parallel, potential sampling bias effects should not be overlooked [53,96] since they may hide a part of disease dynamics such as silent spread in deprived rural areas. Additionally, many endemic countries with high human incidence (Russia, Malaysia, Cambodia, Burma, Niger, Mozambique, etc.) [8] remain largely unstudied using quantitative approaches. This represents an opportunity for data collection, rabies dynamics characterization and control strategy optimization. Besides filling knowledge gaps, improving the availability of epidemiological, ecological, and genetic data offers an opportunity to strengthen countries’ veterinary surveillance capacities [15] and enhance the impact assessment of control strategies, two pillars of the 2030 strategic elimination plan.

**Table 3:** Strengths and weaknesses of phylodynamics and mathematical modelling studies identified in this review for the study of rabies.

	Strengths	Weaknesses
**Phylodynamics**	• Homogeneous methodology which facilitates the comparison of rabies dynamics in different areas • Recent advances in phylogeographical models	• Small datasets and short genetic sequences • Studies generally remain desriptive in terms of environmental factors contributing to rabies spread • Large room to apply other models (such as models implemented in BEAST 2) • The potential impact of reporting biases is barely addressed
**Mathematical modelling**	• Diversity of models that explore multiple aspects of rabies spread • Assessment of rabies control strategies efficacy • Integration of the waning of vaccine-induced immunity	• Mostly simulation studies, models are rarely fitted to dog rabies data • Mostly deterministic models with strong assumptions (homogeneous mixing of dogs, absence of dog population structure, absence of individual heterogeneity) • No direct comparison of rabies dynamics due to the diversity of models

Other data types such as social sciences data could help identify knowledge gaps and refine control measures to be tested further using mathematical models. For example, there is little quantitative evidence of the impact of community response on the efficacy of control measures [91], although it is key to human rabies prevention [97,98] and it is expected to change over rabies outbreaks and affect rabies dynamics. By bridging the two disciplines, alternative control strategies that are both effective and adapted to community preferences could be designed [99] (Fig 4).

Finally, novel methodologies combining genetic, epidemiological, and environmental data in a comprehensive analysis framework are promising tools for the rabies field. Indeed, the interdisciplinary studies identified in this review exploited the complementarity of genetic and epidemiological information to efficiently generate and test hypotheses on the mechanisms of rabies dynamics [20,72,73,76,77], and the limitations of control strategies [74,75]. These new avenues represent a significant improvement on past studies and a promising opportunity for canine rabies research in the frame of a One Health concept that aims to achieve better public health outcomes through cross-sector collaboration.

## Conclusions

In this review, we highlighted the need for more epidemiological, ecological, and genetic data to better characterize rabies dynamics and to get practical information on the drivers of disease transmission. We think that the development of new methodologies integrating genetic and epidemiological data, or the combined use of mathematical models and phylodynamics, constitutes a promising approach that could ultimately contribute to the improvement of the efficacy of control measures including vaccination campaigns and help optimizing the allocation of resources in a context of limited funding.

## Supporting information

S1 Table General characteristics of the included studies. (XLSX)

S2 Table Description of the phylogeographic models with an emphasis on data source and potential sources of bias. (XLSX)

S3 Table Description of the mathematical models with their key quantitative results. (XLSX)

S4 Table Description of the interdisciplinary studies combining phylodynamics and mathematical modelling or integrating epidemiological and genetic data. (XLSX)

S5 Table Detailed list of the estimated parameters in phylodynamic models. (XLSX)

S6 Table Detailed list of the estimated parameters in mathematical models. (XLSX)

S1 Text Rabies epidemiological situation and methodologies implemented to study rabies dispersal and control at the continent level. (PDF)

S1 PRISMA Checklist Preferred Reporting Items for Systematic reviews and Meta-Analyses extension for Scoping Reviews (PRISMA-ScR) Checklist. (PDF)
