# Correction: Mathematical modelling and phylodynamics for the study of dog rabies dynamics and control: A scoping review

**DOI:** 10.1371/journal.pntd.0011155

**Published:** 2023-02-24

**Authors:** Maylis Layan, Simon Dellicour, Guy Baele, Simon Cauchemez, Hervé Bourhy

There is an error in the Data Availability Statement. The Data Availability statement should read as follows: All relevant data are within the manuscript and its Supporting Information files. Supporting Information files are also available online at https://mlayan.github.io/RabiesScopingReview/ and archived on the open-access repository Zenodo (DOI: 10.5281/zenodo.4743553).

On page 9, the third sentence of the penultimate paragraph should cite reference 41 rather than reference 65 in the third clause.

The correct sentence should read: A phylodynamic study at the global scale showed that host shifts from dogs to wildlife with adaptation to the new host were common in RABV history [65], which may explain why different lineages circulate in dogs and wild foxes in Brazil [61], in dogs and ferret badgers in Asia [41] and in dogs and mongooses in South Africa [65] with rare interspecies transmission events.

41. Huang J, Ruan S, Shu Y, Wu X. Modeling the Transmission Dynamics of Rabies for Dog, Chinese Ferret Badger and Human Interactions in Zhejiang Province, China. Bull Math Biol. 2019; 81: 939–962. https://doi.org/10.1007/s11538-018-00537-1 PMID: 30536160

In footnotes a and b of Table 1, the citations of references 67, 68, and 70 should be replaced with references 68, 69, and 71.

The correct sentences in footnote a should read: Brunker et al. [69], Tian et al. [77], and Dellicour et al. [71] estimated the mean branch velocity using continuous phylogeographic reconstructions. Finally, Dellicour et al. [68] estimated the temporal evolution of the wavefront velocity that corresponds to the distance between the reconstructed epidemic origin and the maximal epidemic wavefront. While the mean branch velocity (v) and diffusion coefficient (D) are estimates of the dispersal velocity and of the diffusion coefficient averaged over all tree branches, respectively, their weighted average counterparts involve a weighting by branch time resulting in lower-variance estimates [71].

The correct sentences in footnote b should read: Brunker et al. [69] parametrized a generalized linear model (GLM) in a discrete phylogeographic framework with resistance distances derived from landscape data between clusters of rabies cases. Dellicour et al. [68] and Tian et al. [77] assessed which environmental factors are associated with RABV velocity using continuous phylogeographic inference and post hoc statistical analyses. Dellicour et al. [71] and Tian et al. [77] also identified factors associated with the direction of spread using phylogeographic reconstructions and subsequent post hoc analyses.

68. Dellicour S, Rose R, Faria NR, Vieira LFP, Bourhy H, Gilbert M, et al. Using Viral Gene Sequences to Compare and Explain the Heterogeneous Spatial Dynamics of Virus Epidemics. Mol Biol Evol. 2017; 34: 2563–2571. https://doi.org/10.1093/molbev/msx176 PMID: 28651357

69. Brunker K, Lemey P, Marston DA, Fooks AR, Lugelo A, Ngeleja C, et al. Landscape attributes governing local transmission of an endemic zoonosis: Rabies virus in domestic dogs. Mol Ecol. 2018; 27: 773–788. https://doi.org/10.1111/mec.14470 PMID: 29274171

71. Dellicour S, Troupin C, Jahanbakhsh F, Salama A, Massoudi S, Moghaddam MK, et al. Using phylogeographic approaches to analyse the dispersal history, velocity and direction of viral lineages—Application to rabies virus spread in Iran. Mol Ecol. 2019; 28: 4335–4350. https://doi.org/10.1111/mec.15222 PMID: 31535448

In the footnote of Table 2, the citations of references 35, 40, and 51 should be replaced with references 29, 45, and 34.

The correct sentences should read: Three studies [29,45,34] are not grounded in a specific geographical area. Using simulated scenarios, they test the impact of control strategies according to the time to detection [29], dog population structure [34] and the use of immunocontraceptives [45].

29. Townsend SE, Lembo T, Cleaveland S, Meslin FX, Miranda ME, Putra AAG, et al. Surveillance guidelines for disease elimination: A case study of canine rabies. Comp Immunol Microbiol Infect Dis. 2013; 36: 249–261. https://doi.org/10.1016/j.cimid.2012.10.008 PMID: 23260376

45. Carroll MJ, Singer A, Smith GC, Cowan DP, Massei G. The use of immunocontraception to improve rabies eradication in urban dog populations. Wildl Res. 2010; 37: 676–687. https://doi.org/10.1071/WR10027

34. Leung T, Davis SA. Rabies Vaccination Targets for Stray Dog Populations. Front Vet Sci. 2017; 4: 1–10. https://doi.org/10.3389/fvets.2017.00001 PMID: 28154816

Fig 3 contains incorrect reference numbers. The authors have provided a corrected version of Fig 3 here.

**Fig 3:**
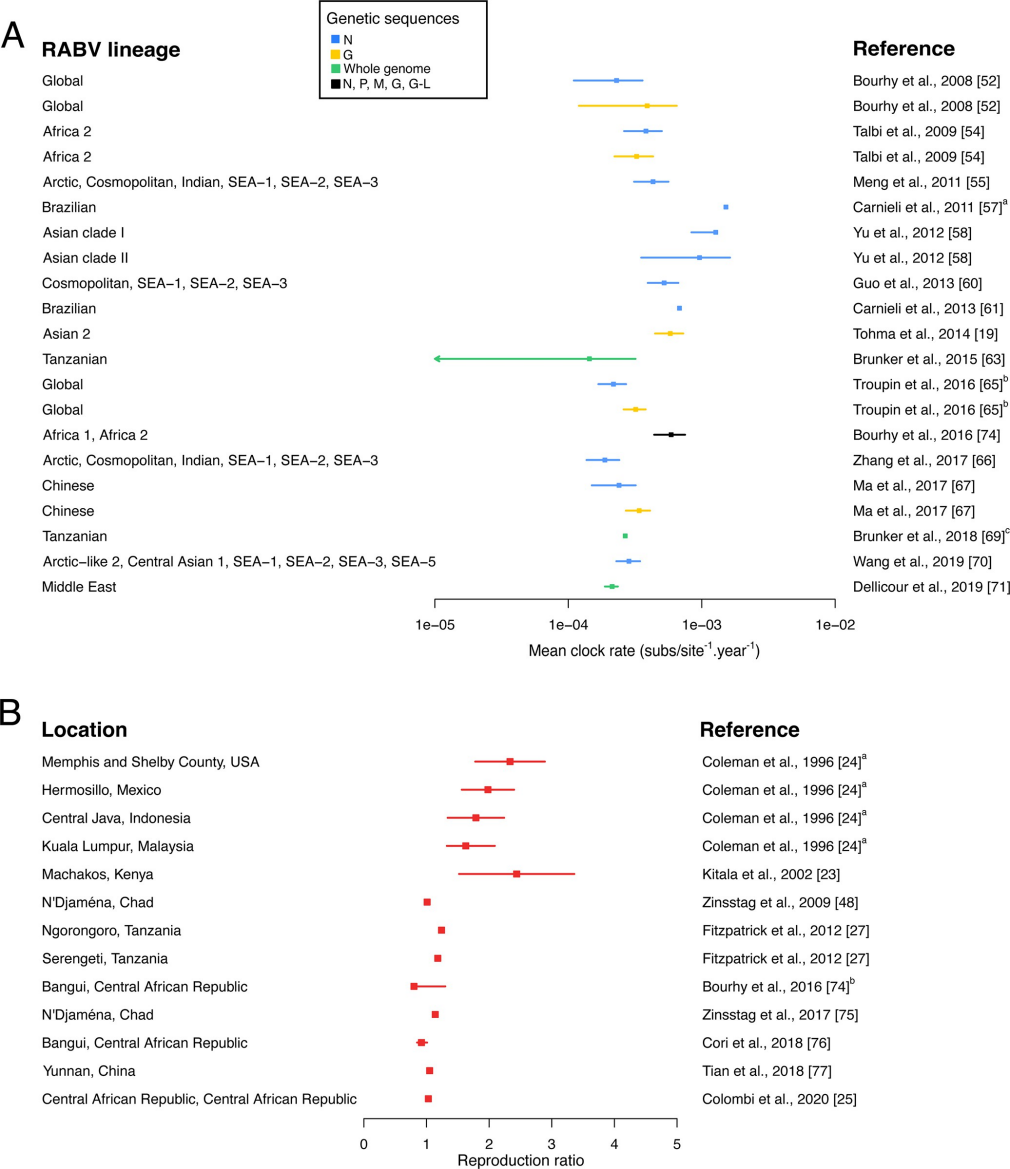
Estimates of the mean evolutionary rate of RABV and the reproduction ratio of canine rabies in the included studies. (A) Bayesian credibility intervals (mean and 95% Highest Posterior Density, HPD) of the mean evolutionary rate of canine RABV per genetic sequence and RABV lineage. aThe estimate corresponds to the upper bound of the 95% HPD. bThe dot corresponds to the median and the interval to the 95% HPD interval. cThe 95% HPD was not specified in the original publication. (B) Estimates of the reproduction ratio of dog rabies per control strategy or geographical location. The dot corresponds to the mean and the interval to the 95% confidence interval unless stated otherwise. a The interval corresponds to the standard error. b The authors estimated the effective reproduction ratio along time. Here, the value range of the median monthly point estimate is depicted.
