# Supplementary material for: Mathematical modelling and phylodynamics for the study of dog rabies dynamics and control: A scoping review
Source: PLoS Negl Trop Dis. 2021 May 27;15(5):e0009449. doi: 10.1371/journal.pntd.0009449 (PMC8189497; doi:10.1371/journal.pntd.0009449)
Supplement: S1 Text — (PDF) [file pntd.0009449.s007.pdf]

Supplementary Text. Rabies epidemiological situation and methodologies implemented to study rabies dispersal and control at the continent level. The situation of North America is not detailed since the study of Coleman & Dye, 1996[1] only estimated the critical vaccination coverage and R from an epidemic in the Tennessee in the 1940s and an epidemic in Mexico in the 1980s.

**Africa**
<u>Current situation:</u><br>Endemic.<br><br><u>Models:</u><br>Large variety with development of multi-host, metapopulation and network models[2,3,12,13,4–11].<br>Parsimony, Bayesian discrete and continuous phylogeography[14–20].<br>Interdisciplinary studies[21–25].<br><br><u>Data:</u><br>Bite incidence in humans, dog, and human rabies incidence, contact tracing, dog mobility data, dog census, RABV genetic sequences from dogs, wildlife, and humans.<br><br><u>Modelling aims:</u><br>Better understanding of the spatial and temporal dynamics of rabies spread[9,12,16–18,23,24].<br>Identification of environmental factors impacting RABV spread and the main patterns of dispersal[5,15,16,19,20,23].<br>Role of introductions[15,24,25], spatial heterogeneity[2,5,6], dog population structure[3,11] and wildlife[6,7] in dog rabies maintenance.<br>Feasible and effective control strategies[4,6–8,10–13,25].<br>Development of new methodologies[21,22].<br>Impact of under-reporting[2,21,25].<br><br><u>Mains findings:</u><br>Rabies spread was studied at multiple geographical scales (transborder area, country, district, city, neighborhood). Studies at small spatial scales supported that local scale elimination is achievable on the short term[2,24,25], but introduction events participate in rabies maintenance[2,6,21,22] and impede control efforts[24,25]. Rabies was shown to circulate at low intensity within two cities, Bangui[24] and N'Djaména[2,12,25], but connections between urban areas are expected to accentuate rabies spread[5]. Indeed, human-mediated movements strongly impact rabies dispersal within countries in North Africa[20,23] and the Central African Republic[5]. According to the setting, they may counteract the effects of control measures.<br>Spatial and individual heterogeneity were not sufficient to explain rabies maintenance in settings with low circulation[2,3] and there is no current evidence of the role of wildlife in the maintenance of rabies[6,7].<br>At the continental scale, there are both co-circulating RABV lineages[14,16–19] and spatial clustering of RABV lineages[14–17,19] which points at the role of human-mediated movements.

The dog vaccination coverage recommended by the WHO (70%) has been generally shown to be sufficient to reach dog rabies elimination[7,12,13], except in Ethiopia where a 90% vaccination coverage was recommended[4]. Vaccination strategies targeting at-risk dog populations are more effective[10]. The role of underreporting is not clear[2,21,25] but heterogeneous vaccination coverage is shown to disrupt vaccination[26].

Asia

Current situation:
Disease-free in Japan.
Recent introductions in Indonesia and Philippines.
Endemic in China and continental South-East Asia.

Models:
Mostly deterministic models implemented to analyze dog rabies in China[27–31], Malaysia[1,32] and Indonesia[1].
Agent-based models[26,33,34].
Phylogeography[35–43].
Interdisciplinary studies[44,45].

Data:
Human rabies cases from passive surveillance, dog rabies cases from active (China) or passive surveillance, contact tracing, dog vaccination data, dog density from surveys, dog movements from household surveys, historical records of dog rabies epidemics in Osaka and of dog and human censuses, RABV genetic sequences from dogs, wildlife and humans.

Modelling aims:
Better understanding of the spatial and temporal dynamics of rabies spread[27,29,41–45,30,32,35–40].
Identification of circulating lineages[38,40,45] and environmental factors impacting rabies spread[43,45].
Spatiotemporal dynamics and interactions of canine and wildlife RABV lineages[31,42].
Feasible and effective control strategies[1,26–29,31,34].
Impact of human-mediated movement[26,34] and vaccination coverage[26] on the efficacy of control strategies.
Modelling dynamics following an introduction and assessment of the efficacy of current contingency plans[33].
Estimation of the time from introduction to detection according to the value of R[44].

Main findings:
Rabies introductions in the disease-free islands of the Philippines result from single introductions from neighboring rabies-endemic islands followed by local transmission[39,44].
At the continental scale, RABV lineages are spatially clustered[37,38] but transboundary movements markedly influence rabies spread[37]. China is endemic for rabies and multiple

RABV lineages co-circulate across the country, notably Asian, Arctic-like and Cosmopolitan lineages[36,42,43]. It is thought to be one of the main sources of RABV lineages in Asia[36,37].
A decade after achieving rabies elimination, it resurged in Yunnan and is currently circulating uncontrolled. This Chinese province corresponds to a crossroads area where multiple RABV lineages circulate, probably resulting from multiple transboundary movements[35,45]. Moreover, rabies dispersal velocity is weakly associated with forest coverage, croplands and accessible areas[45]. Whereas human-mediated movement is not statistically associated with rabies velocity in the Yunnan province[45], it is suspected to have played a role in rabies dispersal in the Shaanxi province[41]. More studies are needed to unravel the interactions between RABV, reservoir ecology and humans in Asia.
In general, rabies is estimated to spread at low grade with an R lower than two[26–32]. Occasional long distance migrations which were documented in the Philippines[44], Indonesia[40] and China[30,43,46] might contribute to disease persistence.
The role of wildlife has been poorly studied and remains unclear in endemic areas[31,42].
Dog vaccination is the most effective strategy[27,28,31,32] and may be improved by complementary measures such as domestic and stray dog management[27–29], dog confinement[34] or increasing public awareness[29,31,33]. Homogeneous vaccination coverage was shown to yield better elimination prospects[8,30,34] which might be due to its robustness to human-mediated movements[8]. In Japan, Kadowaki et al.[33] showed that the current contingency plan is adapted to the rapid detection, control and elimination of rabies after an introduction. The authors emphasized the benefits of dog owner awareness and the control of stray dogs in the improvement of the plan[33].
The time to detection is also a crucial factor in the success of rabies elimination after introduction. The faster the disease is detected, the higher the odds of eradicating it[8]. For example, it’s estimated that the surveillance system detected rabies circulation one year after its introduction in the Luzon island group in the Philippines[44]. This delay would have been greater with a lower reporting capacity[44].

Middle East

Current situation:
Endemic.

Models:
Phylogeography[47,48].

Data:
RABV genetic sequences from dogs, wildlife, and humans.

Modelling aims:
Spatiotemporal dynamics and interactions of canine and wildlife RABV lineages[47,48].
Identification of circulating lineages and environmental factors impacting rabies spread[47].

Main findings:

Many lineages circulate that are phylogenetically related to Asian, Arctic/Artic-like or Cosmopolitan lineages resulting from sustained circulation in dogs and wildlife after introduction[47,48]. There is a strong spatial segregation of RABV lineages circulating in Iran. Overall, their spread is not driven by road connectivity, but humans presumably play a role since lineages tend to disperse towards and remain in highly populated areas. Lineages were less likely to spread towards grasslands and to occur in areas with barren vegetation. These results may be influenced by biased sampling towards populated areas however[47]. Wildlife seems to play a role in rabies maintenance in dog populations[47,48] but data are not sufficiently available to study host shift and dynamics between reservoirs.

South America

Current situation:
Endemic for bat rabies and localized resurgences of rabies in dogs.

Models:
Fuzzy compartmental model[49].
Phylogeography[50,51].

Data:
Serological data and RABV genetic sequences from dogs and wildlife.

Modelling aims:
Implementation of a fuzzy logic approach to model rabies spread[49].
Spatiotemporal dynamics of wild fox[50] and dog[50,51] RABV lineages.

Main findings:
Despite extensive dog vaccination campaigns, multiple dog-related RABV lineages circulate in Brazil with a relatively recent common ancestor estimated in the 1950s[50,51]. Dog lineages are generally spatially clustered[50,51] and lineages circulating in wild foxes and dogs are phylogenetically and dynamically independent[50].

Oceania

Current situation:
Rabies-free.

Models:
Agent-based[52–55] and compartmental[56] models.

Data:
Dog population structure, dog roaming behavior (GPS data, questionnaires/interviews of dog owners), dog contacts, census data.

Modelling aims:
Modelling dynamics following an introduction[52–56] and assessment of the most effective control strategies[52,53,55,56].

Main findings:
Australian studies focused on rabies spread in remote rural and peri-urban locations where rabies is expected to be introduced and where surveillance systems might be weakened by the remoteness.
Rabies dynamics are expected to differ between dog categories, such as explorer dogs, roaming dogs or domestic dogs[54–56], and consequently between rural and peri-urban areas[56].
Reactive vaccination after the detection of rabies introduction is the only beneficial strategy[52,53,55,56]. A 90% dog vaccination coverage is recommended to break down rabies spread[55,56] and targeting at-risk dogs should enhance vaccination campaigns efficacy[53,56].

References

1. Coleman PG, Dye C. Immunization coverage required to prevent outbreaks of dog rabies. Vaccine. 1996;14: 185–186. doi:10.1016/0264-410X(95)00197-9
2. Laager M, Léchenne M, Naissengar K, Mindekem R, Oussiguere A, Zinsstag J, et al. A metapopulation model of dog rabies transmission in N'Djamena, Chad. J Theor Biol. 2019;462: 408–417. doi:10.1016/j.jtbi.2018.11.027
3. Wilson-Aggarwal JK, Ozella L, Tizzoni M, Cattuto C, Swan GJF, Moundai T, et al. High-resolution contact networks of free-ranging domestic dogs Canis familiaris and implications for transmission of infection. PLoS Negl Trop Dis. 2019;13: 1–19. doi:10.1371/journal.pntd.0007565
4. Beyene TJ, Fitzpatrick MC, Galvani AP, Mourits MCM, Revie CW, Cernicchiaro N, et al. Impact of One-Health framework on vaccination cost-effectiveness: A case study of rabies in Ethiopia. One Heal. 2019;8: 100103. doi:10.1016/j.onehlt.2019.100103
5. Colombi D, Poletto C, Nakouné E, Bourhy H, Colizza V. Long-range movements coupled with heterogeneous incubation period sustain dog rabies at the national scale in Africa. PLoS Negl Trop Dis. 2020;14: e0008317. doi:10.1371/journal.pntd.0008317
6. Beyer HL, Hampson K, Lembo T, Cleaveland S, Kaare M, Haydon DT. Metapopulation dynamics of rabies and the efficacy of vaccination. Proc R Soc B Biol Sci. 2011;278: 2182–2190. doi:10.1098/rspb.2010.2312
7. Fitzpatrick MC, Hampson K, Cleaveland S, Meyers LA, Townsend JP, Galvani AP. Potential for Rabies Control through Dog Vaccination in Wildlife-Abundant Communities of Tanzania. PLoS Negl Trop Dis. 2012;6. doi:10.1371/journal.pntd.0001796
8. Townsend SE, Lembo T, Cleaveland S, Meslin FX, Miranda ME, Putra AAG, et al. Surveillance guidelines for disease elimination: A case study of canine rabies. Comp Immunol Microbiol Infect Dis. 2013;36: 249–261. doi:10.1016/j.cimid.2012.10.008
9. Hampson K, Dushoff J, Bingham J, Brückner G, Ali YH, Dobson A. Synchronous cycles of domestic dog rabies in sub-Saharan Africa and the impact of control efforts. Proc Natl Acad Sci U S A. 2007;104: 7717–7722. doi:10.1073/pnas.0609122104
10. Beyer HL, Hampson K, Lembo T, Cleaveland S, Kaare M, Haydon DT. The implications of metapopulation dynamics on the design of vaccination campaigns. Vaccine. 2012;30: 1014–1022. doi:10.1016/j.vaccine.2011.12.052
11. Laager M, Mbilo C, Madaye EA, Naminou A, Léchenne M, Tschopp A, et al. The importance of dog population contact network structures in rabies transmission. PLoS

Negl Trop Dis. 2018;12: 1–18. doi:10.1371/journal.pntd.0006680
12. Zinsstag J, Dürr S, Penny MA, Mindekem R, Roth F, Menendez Gonzalez S, et al. Transmission dynamics and economics of rabies control in dogs and humans in an African city. Proc Natl Acad Sci U S A. 2009;106: 14996–15001. doi:10.1073/pnas.0904740106
13. Kitala PM, McDermott JJ, Coleman PG, Dye C. Comparison of vaccination strategies for the control of dog rabies in Machakos District, Kenya. Epidemiol Infect. 2002;129: 215–222. doi:10.1017/S0950268802006957
14. Lemey P, Rambaut A, Drummond AJ, Suchard MA. Bayesian phylogeography finds its roots. PLoS Comput Biol. 2009;5. doi:10.1371/journal.pcbi.1000520
15. Mollentze N, Weyer J, Markotter W, Le Roux K, Nel LH. Dog rabies in southern Africa: Regional surveillance and phylogeographical analyses are an important component of control and elimination strategies. Virus Genes. 2013;47: 569–573. doi:10.1007/s11262-013-0974-3
16. Brunker K, Marston DA, Horton DL, Cleaveland S, Fooks AR, Kazwala R, et al. Elucidating the phylodynamics of endemic rabies virus in eastern Africa using whole-genome sequencing. Virus Evol. 2015;1: 1–11. doi:10.1093/ve/vev011
17. Talbi C, Holmes EC, de Benedictis P, Faye O, Nakouné E, Gamatié D, et al. Evolutionary history and dynamics of dog rabies virus in western and central Africa. J Gen Virol. 2009;90: 783–791. doi:10.1099/vir.0.007765-0
18. Hayman DTS, Johnson N, Horton DL, Hedge J, Wakeley PR, Banyard AC, et al. Evolutionary history of rabies in Ghana. PLoS Negl Trop Dis. 2011;5. doi:10.1371/journal.pntd.0001001
19. Brunker K, Lemey P, Marston DA, Fooks AR, Lugelo A, Ngeleja C, et al. Landscape attributes governing local transmission of an endemic zoonosis: Rabies virus in domestic dogs. Mol Ecol. 2018;27: 773–788. doi:10.1111/mec.14470
20. Dellicour S, Rose R, Faria NR, Vieira LFP, Bourhy H, Gilbert M, et al. Using Viral Gene Sequences to Compare and Explain the Heterogeneous Spatial Dynamics of Virus Epidemics. Mol Biol Evol. 2017;34: 2563–2571. doi:10.1093/molbev/msx176
21. Mollentze N, Nel LH, Townsend S, le Roux K, Hampson K, Haydon DT, et al. A bayesian approach for inferring the dynamics of partially observed endemic infectious diseases from space-time-genetic data. Proc R Soc B Biol Sci. 2014;281. doi:10.1098/rspb.2013.3251
22. Cori A, Nouvellet P, Garske T, Bourhy H, Nakouné E, Jombart T. A graph-based evidence synthesis approach to detecting outbreak clusters: An application to dog rabies. PLoS Comput Biol. 2018;14. doi:10.1371/journal.pcbi.1006554
23. Talbi C, Lemey P, Suchard MA, Abdelatif E, Elharrak M, Jalal N, et al. Phylodynamics and Human-mediated dispersal of a zoonotic virus. PLoS Pathog. 2010;6. doi:10.1371/journal.ppat.1001166
24. Bourhy H, Nakouné E, Hall M, Nouvellet P, Lepelletier A, Talbi C, et al. Revealing the Micro-scale Signature of Endemic Zoonotic Disease Transmission in an African Urban Setting. PLoS Pathog. 2016;12: e1005525. doi:10.1371/journal.ppat.1005525
25. Zinsstag J, Lechenne M, Laager M, Mindekem R, Naïssengar S, Oussiguéré A, et al. Vaccination of dogs in an African city interrupts rabies transmission and reduces human exposure. Sci Transl Med. 2017;9. doi:10.1126/scitranslmed.aaf6984
26. Townsend SE, Sumantra IP, Pudjiatmoko, Bagus GN, Brum E, Cleaveland S, et al. Designing Programs for Eliminating Canine Rabies from Islands: Bali, Indonesia as a Case Study. PLoS Negl Trop Dis. 2013;7. doi:10.1371/journal.pntd.0002372
27. Zhang J, Jin Z, Sun GQ, Zhou T, Ruan S. Analysis of rabies in China: transmission dynamics and control. PLoS One. 2011;6. doi:10.1371/journal.pone.0020891
28. Hou Q, Jin Z, Ruan S. Dynamics of rabies epidemics and the impact of control efforts in Guangdong Province, China. J Theor Biol. 2012;300: 39–47. doi:10.1016/j.jtbi.2012.01.006
29. Zhang J, Jin Z, Sun GQ, Sun XD, Ruan S. Modeling Seasonal Rabies Epidemics in China. Bull Math Biol. 2012;74: 1226–1251. doi:10.1007/s11538-012-9720-6

30. Chen J, Zou L, Jin Z, Ruan S. Modeling the Geographic Spread of Rabies in China. PLoS Negl Trop Dis. 2015;9: 1–18. doi:10.1371/journal.pntd.0003772
31. Huang J, Ruan S, Shu Y, Wu X. Modeling the Transmission Dynamics of Rabies for Dog, Chinese Ferret Badger and Human Interactions in Zhejiang Province, China. Bull Math Biol. 2019;81: 939–962. doi:10.1007/s11538-018-00537-1
32. Taib NAA, Labadin J, Piau P. Model simulation for the spread of rabies in Sarawak, Malaysia. Int J Adv Sci Eng Inf Technol. 2019;9: 1739–1745. doi:10.18517/ijaseit.9.5.10230
33. Kadowaki H, Hampson K, Tojinbara K, Yamada A, Makita K. The risk of rabies spread in Japan: a mathematical modelling assessment. Epidemiol Infect. 2018;146: 1245–1252. doi:10.1017/s0950268818001267
34. Ferguson EA, Hampson K, Cleaveland S, Consunji R, Deray R, Friar J, et al. Heterogeneity in the spread and control of infectious disease: Consequences for the elimination of canine rabies. Sci Rep. 2015;5: 1–13. doi:10.1038/srep18232
35. Zhang Y, Vrancken B, Feng Y, Dellicour S, Yang Q, Yang W, et al. Cross-border spread, lineage displacement and evolutionary rate estimation of rabies virus in Yunnan Province, China. Virol J. 2017;14: 1–8. doi:10.1186/s12985-017-0769-6
36. Meng S, Sun Y, Wu X, Tang J, Xu G, Lei Y, et al. Evolutionary dynamics of rabies viruses highlights the importance of China rabies transmission in Asia. Virology. 2011;410: 403–409. doi:10.1016/j.virol.2010.12.011
37. Guo Z, Tao X, Yin C, Han N, Yu J, Li H, et al. National Borders Effectively Halt the Spread of Rabies: The Current Rabies Epidemic in China Is Dislocated from Cases in Neighboring Countries. PLoS Negl Trop Dis. 2013;7. doi:10.1371/journal.pntd.0002039
38. Wang L, Wu X, Bao J, Song C, Du J. Phylodynamic and transmission pattern of rabies virus in China and its neighboring countries. Arch Virol. 2019. doi:10.1007/s00705-019-04297-8
39. Tohma K, Saito M, Kamigaki T, Tuason LT, Demetria CS, Orbina JRC, et al. Phylogeographic analysis of rabies viruses in the Philippines. Infect Genet Evol. 2014;23: 86–94. doi:10.1016/j.meegid.2014.01.026
40. Dibia IN, Sumiarto B, Susetya H, Putra AAG, Scott-Orr H, Mahardika GN. Phylogeography of the current rabies viruses in Indonesia. J Vet Sci. 2015;16: 459–466. doi:10.4142/jvs.2015.16.4.459
41. Ma C, Hao X, Deng H, Wu R, Liu J, Yang Y, et al. Re-emerging of rabies in Shaanxi province, China, from 2009 to 2015. J Med Virol. 2017;89: 1511–1519. doi:10.1002/jmv.24769
42. Yu J, Li H, Tang Q, Rayner S, Han N, Guo Z, et al. The spatial and temporal dynamics of rabies in China. PLoS Negl Trop Dis. 2012;6. doi:10.1371/journal.pntd.0001640
43. Yao HW, Yang Y, Liu K, Li X Lou, Zuo SQ, Sun RX, et al. The Spatiotemporal Expansion of Human Rabies and Its Probable Explanation in Mainland China, 2004-2013. PLoS Negl Trop Dis. 2015;9: 2004–2013. doi:10.1371/journal.pntd.0003502
44. Tohma K, Saito M, Demetria CS, Manalo DL, Quiambao BP, Kamigaki T, et al. Molecular and mathematical modeling analyses of inter-island transmission of rabies into a previously rabies-free island in the Philippines. Infect Genet Evol. 2016;38: 22–28. doi:10.1016/j.meegid.2015.12.001
45. Tian H, Feng Y, Vrancken B, Cazelles B, Tan H, Gill MS, et al. Transmission dynamics of re-emerging rabies in domestic dogs of rural China. PLoS Pathog. 2018;14: 1–18. doi:10.1371/journal.ppat.1007392
46. Guo D, Zhou H, Zou Y, Yin W, Yu H, Si Y, et al. Geographical Analysis of the Distribution and Spread of Human Rabies in China from 2005 to 2011. PLoS One. 2013;8: 1–10. doi:10.1371/journal.pone.0072352
47. Dellicour S, Troupin C, Jahanbakhsh F, Salama A, Massoudi S, Moghaddam MK, et al. Using phylogeographic approaches to analyse the dispersal history, velocity and direction of viral lineages — Application to rabies virus spread in Iran. Mol Ecol. 2019;28: 4335–4350. doi:10.1111/mec.15222
48. Horton DL, McElhinney LM, Freuling CM, Marston DA, Banyard AC, Goharrriz H, et al.

Complex Epidemiology of a Zoonotic Disease in a Culturally Diverse Region: Phylogeography of Rabies Virus in the Middle East. PLoS Negl Trop Dis. 2015;9: 1–17. doi:10.1371/journal.pntd.0003569
49. Ortega NRS, Sallum PC, Massad E. Fuzzy dynamical systems in epidemic modeling. Stud Fuzziness Soft Comput. 2000;232: 181–206. doi:10.1007/978-3-540-69094-8_9
50. Carnieli P, Ruthner Batista HBC, de Novaes Oliveira R, Castilho JG, Vieira LFP. Phylogeographic dispersion and diversification of rabies virus lineages associated with dogs and crab-eating foxes (Cerdocyon thous) in Brazil. Arch Virol. 2013;158: 2307–2313. doi:10.1007/s00705-013-1755-y
51. Carnieli P, de Novaes Oliveira R, Macedo CI, Castilho JG. Phylogeography of rabies virus isolated from dogs in Brazil between 1985 and 2006. Arch Virol. 2011;156: 1007–1012. doi:10.1007/s00705-011-0942-y
52. Dürr S, Ward MP. Development of a novel rabies simulation model for application in a non-endemic environment. PLoS Negl Trop Dis. 2015;9: 1–22. doi:10.1371/journal.pntd.0003876
53. Hudson EG, Brookes VJ, Dürr S, Ward MP. Modelling targeted rabies vaccination strategies for a domestic dog population with heterogeneous roaming patterns. PLoS Negl Trop Dis. 2019;13: 1–15. doi:10.1371/journal.pntd.0007582
54. Hudson EG, Brookes VJ, Ward MP, Dürr S. Using roaming behaviours of dogs to estimate contact rates: The predicted effect on rabies spread. Epidemiol Infect. 2019;147. doi:10.1017/S0950268819000189
55. Brookes VJ, Dürr S, Ward MP. Rabies-induced behavioural changes are key to rabies persistence in dog populations: Investigation using a network-based model. PLoS Negl Trop Dis. 2019;13: 1–19. doi:10.1371/journal.pntd.0007739
56. Sparkes J, McLeod S, Ballard G, Fleming PJS, Körtner G, Brown WY. Rabies disease dynamics in naïve dog populations in Australia. Prev Vet Med. 2016;131: 127–136. doi:10.1016/j.prevetmed.2016.07.015
